# The National Hospital for the Paralysed and Epileptic

**Published:** 1900-08-25

**Authors:** 


### THE NATIONAL HOSPITAL FOR THE PARALYSED AND EPILEPTIC.

Referring to the recent meeting of the Governors of this charity, which was reported last week in The Hospital, the *British Medical Journal* says that the situation is that a handful of Governors have decided to refuse to allow the medical staff, without whom the hospital could not be carried on for a day, any voice in its management, and have apparently accepted the suggestion of the gentleman who presided over the inquiry into the condition of the hospital that, as he is satisfied with it, they should accept it as conclusive. The *British Medical Journal* submits that something more than this is required, and that Mr. Russell is grievously mistaken if he fancies that this is to be the end of the matter. The *Lancet* is still more severe, saying that a special committee appointed by the Board of Management was not the proper body to report on the matter, for the Board of Management was itself the accused party, and cannot seriously constitute itself its own judge. Turning to the points at issue it says that the medical staff of this hospital are capable citizens, as well as distinguished neurologists, while the secretary-director is the salaried servant of the hospital, and that it is not right that he should be the only, or even the principal, medium between them and the Board of Management. The medical staff have the right to know that what they say and what they wish, exactly as they say it and wish it, reaches the ears of the Board of Management, and there should be no suspicion that only such matters as are deemed by a salaried official to be worth the attention of the board are laid before the board. Everything that the medical staff have to say must be heard, and they must be the judges of whether it is worth hearing, and the obviously easy way of accomplishing this is for the board to include representatives of the medical staff. As to the vivisection bogie and the "horror" entertained by the working man of entering hospitals, the *Lancet* puts it on one side, agreeing with Sir Henry Burdett that, greatly as the number of beds have been increased, for the thousands of beds there are always tens of thousands of applicants. So that side of the matter need not be discussed.

In the *Times* of the 21st inst. a long and forcible letter appears from Mr. Brudenell Carter, who, although he writes unofficially, no doubt voices the feeling of the medical staff in much that he says. He narrows the question down to the point of representation of the staff on the board, and he ends by saying:—

"The case lies in a nutshell. A proper reform of the constitution of the hospital would leave it in the very van of curative work in the growing science of neurology; while a victory for the board would reduce it to a receptacle of incurable paralytics and hysterical impostors, living in an establishment controlled by a secretary-director, and nominally under the medical care of weak or incompetent practitioners."

In the same issue of the *Times* there is also a letter from Sir Henry Burdett primarily in reference to the action of the Hospital Sunday Fund. In it, however, he makes the following important statement in regard to the real root of the matter:—

"It came out at the governors' meeting that, instead of the staff having an opportunity to discuss all matters directly with members of the Board of Management, as provided by No. 1 of the bye-laws regulating the action of the Medical Committee, the channel of communication of late years had come to be the secretary-director; and the staff felt, not unnaturally, that they had first to convert this gentleman to their views and then the Board of Management, or nothing could be carried through. In these circumstances and in course of time such a system was bound to break down, as it has done at this hospital. I am quite confident that it should not be difficult to build a golden bridge whereby a satisfactory way out of the difficulty may be speedily found."

